# Inhibition of *Escherichia coli* and *Bacillus subtilis* FtsZ Polymerization and *Bacillus subtilis* Growth by Dihydroxynaphtyl Aryl Ketones

**DOI:** 10.3389/fmicb.2019.01225

**Published:** 2019-06-12

**Authors:** Gissela Araya, Julio Benites, Juan S. Reyes, Andrés E. Marcoleta, Jaime A. Valderrama, Rosalba Lagos, Octavio Monasterio

**Affiliations:** ^1^Departamento de Biología, Facultad de Ciencias, Universidad de Chile, Santiago, Chile; ^2^Facultad de Ciencias de la Salud, Universidad Arturo Prat, Iquique, Chile; ^3^Instituto de EtnoFarmacología (IDE), Universidad Arturo Prat, Iquique, Chile

**Keywords:** diaryl-ketones, bacterial inhibitors, FtsZ, polymerization, *E. coli*, *B. subtilis*

## Abstract

The increasing detection of virulent and/or multidrug resistant bacterial strains makes necessary the development of new antimicrobial agents acting through novel mechanisms and cellular targets. A good choice are molecules aimed to interfere with the cell division machinery or *divisome*, which is indispensable for bacterial survival and propagation. A key component of this machinery, and thus a good target, is FtsZ, a highly conserved GTPase protein that polymerizes in the middle of the cell on the inner face of the cytoplasmic membrane forming the Z ring, which acts as a scaffold for the recruitment of the *divisome* proteins at the division site. In this work, we tested the inhibitory effect of five diaryl naphtyl ketone (dNAK) molecules on the *in vitro* polymerization of both *Escherichia coli* and *Bacillus subtilis* FtsZ (EcFtsZ and BsFtsZ, respectively). Among these compounds, dNAK 4 showed the strongest inhibition of FtsZ polymerization *in vitro*, with an IC_50_ of 2.3 ± 0.06 μM for EcFtsZ and 9.13 ± 0.66 μM for BsFtsZ. We found that dNAK 4 binds to GDP-FtsZ polymers but not to the monomer in GTP or GDP state. This led to the polymerization of short and curved filaments, rings, open rings forming clusters, and in the case of BsFtsZ, a novel cylindrical structure of stacked open rings. *In vivo*, dNAK 4 had almost no effect on the growth of *E. coli* in liquid culture, in contrast to the strong inhibitory effect observed over *B. subtilis* growth. The insensitivity of *E. coli* to this compound is probably related to the impermeability of dNAK 4 to the outer membrane. The low amount of this compound required to inhibit several of the bacterial strains tested and the lack of a cytotoxic effect at the concentrations used, makes dNAK 4 a very good candidate as a starting molecule for the development of a new antibiotic.

## Introduction

Infections with multi-resistant bacterial strains have become a worldwide public health problem that urgently needs the development of new antibiotics. Although different strategies to combat or prevent infections have been developed during the last century, including the use of vaccines or antimicrobial chemotherapy, the frequent detection of pathogenic bacteria displaying resistance to multiple antibiotics is currently a major concern for public health. The resistance has increased significantly during the last 25 years, as shown by the increment in the incidence of multi resistant *Staphylococcus aureus* (MRSA), the resistance to vancomycins in *Enterococci* and fluoroquinolones in *Pseudomonas* infections, among several other cases (Walsh and Wencewicz, 2016). So far, the development of resistance by bacteria is inevitable, which makes urgent the development of new antibiotics with new mechanisms of action and novel targets (Morens et al., 2004; Walsh and Wencewicz, 2016).

In this regard, a very good target for the development of antibiotics is FtsZ, the first protein recruited to the division site in bacteria that forms the so-called Z-ring (reviewed in den Blaauwen et al., 2014). This ring is the scaffold for the formation of the division machinery, called divisome, at the division site (Vicente and Rico, 2006). At least 30 compounds have been described as inhibitors of FtsZ (den Blaauwen et al., 2014) and they have been classified into seven groups according to their structure, of which those containing an indole derivative group appear to be the most effective (Anderson et al., 2012). However, the specificity of action of several of these compounds over FtsZ on bacterial division has been questioned (Foss et al., 2011), which would significantly reduce the number of compounds that target FtsZ and that could be used as safe antibiotics. In spite of this, within the heterocyclic group of compounds, PC190723 stands out for its inhibitory properties on bacterial growth, through the stabilization of Z-ring filaments affecting its dynamics (Schaffner-Barbero et al., 2012). The molecular mechanism for the action of this compound is similar to that of taxol over tubulin polymerization, supporting the high structural similarity between FtsZ and tubulin (Andreu et al., 2010). The design of some FtsZ inhibitors has been based on the “antitubulin approach,” as described by Lock and Harry (2008). In a similar way, we synthesized dihydroxynaphthyl ketones with different heterocycles called diaryl naphtyl ketone (dNAKs), analogs of colchicine and naphtyl phenstatins, as potential inhibitors of tubulin polymerization (Benites et al., 2011). Among them, five dNAKs were good inhibitors of tubulin polymerization (Gutiérrez et al., 2015), and its potential use as antitumor agents was evaluated using cancer cells from prostate, bladder, and breast (Benites et al., 2016). The best inhibitor for *in vitro* tubulin polymerization was [(1,4-dihydroxy-naphthalen-2-yl)-(1*H*-pyrrol-2-yl)-methanone] (dNAK 4 in this work) with a Kd of 1.02 ± 0.23 μM, although it showed no effect on normal and cancer cell proliferation. In this work, we evaluated the inhibition of *Escherichia coli* FtsZ polymerization by the dNAK derivatives, being dNAK 4 the best inhibitor. This result and the lack of a cytotoxic effect of dNAK 4 on animal cells (Benites et al., 2016) makes this compound a good candidate for studying its use as a potential antibiotic. We characterized the effect of dNAK 4 on the *in vitro* assembly of FtsZ filaments using purified FtsZ from *E. coli* and *Bacillus subtilis*, as well as the effect of this compound on the growth of both bacterial strains. In addition, using bioinformatics approaches, we characterized the binding site of dNAK 4 to EcFtsZ.

## Materials and Methods

### Chemicals

Tetramethylethylenediamine (TEMED), sodium dodecyl sulfate (SDS), guanosine-5′-triphosphate (GTP), guanosine-5′-diphosphate (GDP), ethylenediaminetetraacetic acid (EDTA), ammonium persulfate, dimethyl sulfoxide (DMSO), bovine serum albumin (BSA), rabbit muscle piruvate kinase (PK), sodium salt glutamate, comassie R250, 2- [fosfonoxil] 2-propenoic acid monocyclohexylammonium salt (PEP), pyruvate (sodium salt), and spectinomycin, were purchased from Sigma Aldrich Chem. Co. (Missouri, USA). Ampicilin, magnesium chloride, calcium chloride, potassium chloride, ammonium sulfate, isopropyl alcohol, hydrochloric acid, glycerol, dimethylformamide, glutaraldehyde, and pro analysis ethanol were purchased from Merck (Darmstadt, Germany). Tween 20 and glycerol were purchased from Winkler (Santiago, Chile). Acetic acid, ethanol and technical grade methanol were obtained from TCL (Santiago, Chile). LB culture medium was purchased from Mo Bio Laboratories (California, USA). Agar, MH (Müller-Hinton) culture medium and Gram staining kit were obtained from Difco laboratories (Michigan, USA). 4-(2-Hydroxyethyl)piperazine-1-ethanesulfonic acid sodium salt (HEPES) and isopropyl β-D-1-thiogalactopyranoside (IPTG) were obtained from US Biological (Massachusetts, USA). Tris(hydroxymethyl)aminomethane (TRIS) was obtained from Amresco Inc (Ohio, USA). 2-(*N*-morpholino) ethanesulfonic acid (MES) was acquired at Phyto Technology laboratories (Kansas, USA). The 40% acrylamide/bis (37.5:1) solution was obtained from BIORAD (California, USA). The dialysis membranes were obtained from Spectrum labs (Ontario, Canada). The 400 mesh carbon-coated copper electron microscopy grids were obtained from Ted Pella, Inc. Uranyl acetate obtained from Riedel de Haën (Hanover, Germany). In the experiments, we used nanopure water (resistivity > 17 MΩ-cm).

### dNAKs

Compounds 1–4 were prepared from 1,4-naphthoquinone and the corresponding aldehydes: thiophene-2-carbaldehyde; thiophene-3-carbaldehyde; furan-2-carbaldehyde; and 1H-pyrrole-2-carbaldehyde (Benites et al., 2011). The structure of compounds 1–4 was confirmed by ^1^H, ^13^C NMR spectra (Arenas et al., 2013). Compound 5 was prepared from 1,4-naphthoquinone and 3,4,5-trimethoxybenzaldehyde, and the spectral data were in agreement with those reported in the literature (Iribarra et al., 2012). The dNAKs were purified by means of flash chromatography on silica gel, eluting with ethyl acetate/petroleum ether, until no trace of impurity (<1%) was detected by ^1^H NMR at 400 MHz. Also, the purity of the dNAKs was checked by reverse phase HPLC chromatography at 220 nm showing purity for dNAKs 1, 2, 3, 4, and 5 of 96, 96, 93, 98, and 95%, respectively.

The absorption spectra of the compounds in DMSO, between 190 and 820 nm, was measured in a Hewlett-Packard 8452A diode array spectrophotometer. For each maximum of the spectrum the molar extinction coefficient was calculated. The maximum solubility in polymerization buffer and the stability in DMSO of the compounds were determined by measuring the absorbance spectra. For each experiment, the maximal concentration used corresponding to the maximal solubility of the compound. Fresh DMSO solutions of the compounds were prepared daily.

### Partition Coefficient Assay

Octanol-water partition coefficient (LogP) values were calculated for all compounds as described by Benites et al. (2012).

### Protein Purification

The EcFtsZ protein was overexpressed in the *E. coli* BL21 (DE3) strain, transformed with the vector PMFV57, which provides a transcription system of the inducible *fts*Z gene by IPTG and presents resistance to ampicillin. The BsFtsZ protein was overexpressed in *E. coli* DH10B strain, transformed with the vectors pCXZ and pBS58. The IPTG-inducible pCXZ vector corresponds to a high copy number plasmid expressing *Bsfts*Z gene and resistance to ampicillin. The vector pBS58 corresponds to a plasmid of very low copy number that expresses the *fts*QAZ genes of *E. coli*, necessary for the viability of the bacteria during the overexpression of BsFtsZ, giving resistance to spectinomycin (Wang and Lutkenhaus, 1993). Cells were grown at 37°C with orbital agitation in 4 L of LB broth containing the respective antibiotics until an OD_600_ of 0.6 was reached, then 0.5 mM IPTG was added and the cells were grown for an additional 3 h. The cells were harvested by centrifugation in a Sorvall centrifuge at 7.000 × g for 10 min, the pellet was suspended in buffer A (50 mM Tris-HCl buffer pH 8.0, 50 mM KCl, 1 mM EDTA, and 5% glycerol) for EcFtsZ and buffer TKM (50 mM Tris-HCl buffer pH 8.0, 50 mM KCl, 1 mM EDTA, 5 mM MgCl_2_, and 5% glycerol) for BsFtsZ, and lysed by sonication (6 cycles of 20 s at 6.0 watts followed by a pause of 20 s). The lysed cells were centrifuged at 100,000 × g for 90 min and the supernatant was saturated with 30% ammonium sulfate and centrifuged at 10.000 × g at 4°C. The pellet was dissolved in buffer A (or TKM), and then dialyzed against 2 L of buffer A (TKM) at 4°C to remove the excess of salt. The next step involved FtsZ polymerization cycles based on the procedure described by Montecinos-Franjola et al. (2012). Briefly, three monosodium glutamate polymerization and depolymerization cycles were performed in 50 mM MES (pH 7.3), 1 M glutamate, 10 mM CaCl_2_, 10 mM MgCl_2_, and 2 mM GTP for 30 min at 37°C, and the polymers were centrifuged at 10,000 × g for 30 min at 25°C. The protein was solubilized in buffer A (TKM) and dialyzed to remove the nucleotide excess. The protein concentration was determined by the Bradford method calibrated for FtsZ. The total protein concentration after purification was typically 15–20 mg mL^−1^ for EcFtsZ and 8–10 mg ml^−1^ for BsFtsZ.

### Polymerization Measurements

Polymerization of FtsZ was determined by light scattering. Light scattering was measured on a Perkin Elmer LS 50 spectrofluorimeter, illuminating with light at a wavelength of 350 nm (excitation) and recording at the same wavelength (emission). Excitation and emission bandwidth were 7 nm and a 4% transmittance filter was used in the emission path. Temperature was kept constant at 30°C. The absorbance at 350 nm of dNAK 4 remained unchanged during the time of the experiments, indicating that the compound was not damaged by the excitation light.

#### GTP Induced Polymerization

Polymerization of FtsZ induced by GTP [with GTP regeneration system PK/PEP (Margalit et al., 2004)] was measured in the absence and the presence of dNAK 4. 12.5 μM FtsZ was pre-incubated in 50 mM HEPES pH 7.3, 50 mM KCl, 10 mM MgCl_2_, 1% DMSO, and 1 mM PEP. The reaction was started by adding the compound (at the concentration indicated in the figures), 4 units of PK and 1 mM GTP. For each polymerization reaction, a baseline was registered, which corresponds to the light scattering after adding the compound and prior to the induction of polymerization by the addition of GTP and PK.

#### GDP Induced Polymerization

Polymerization of FtsZ induced by GDP was measured in the absence and the presence of dNAK 4. 12.5 μM FtsZ was pre-incubated in 50 mM HEPES pH 7.3, 50 mM KCl, 10 mM MgCl_2_, 1% DMSO, and 2 mM GDP. The reaction was started by adding the compound at 32 μM final concentration.

### Electron Microscopy

FtsZ polymerization products were negatively stained and visualized using transmission electron microscopy. Samples of 10 μL were taken during the steady state of GTP and GDP polymerization. The sample was deposited on a grid, previously activated for 30 min with near UV light, and left resting for 1 min. The excess sample was removed with filter paper, then washed with 10 μL of nanopure water and the water excess was removed from the grid with filter paper. To stain, the grids were incubated in 2% uranyl acetate for 1 min, removing the excess with filter paper. To visualize the polymers, a transmission electron microscope PHILIPS TECNAI 12 BIOTWIN of the UMA, PUC was used. The dimensional analysis of the filaments was done with the ImageJ software (http://imagej.nih.gov/ij/).

### *E. coli* and *B. subtilis* Growth Curves

Cells were grown in liquid culture medium Müller Hinton (MH) in the presence and absence of the compound. MH medium was inoculated with an overnight culture of each strain and grown until an OD_600_ of 0.6 was reached. Both cultures were then diluted to OD_600_ of 0.1 and subsequently divided into several cultures, where the controls (without the compound and with DMSO) and different concentrations of dNAK 4 were tested. Each experimental condition was assayed in 250 μL, six times, in a 96-well ELISA plate. The cultures were grown under agitation at 37°C in a plate spectrofluorimeter with absorbance module model TECAN Infinite M200 PRO and the OD_600_ was automatically registered every 15 min for 24 h.

### Molecular Docking

The dNAK 4 binding site in EcFtsZ was modeled by bioinformatics with the Autodock Vina software (Trott and Olson, 2010) was used on a 2.4 GHz *Intel Core 2 Quad* with 4 GB RAM. A model of the EcFtsZ monomer was generated using the amino acid sequence of EcFtsZ (FTSZ_ECOLI) and the crystal structures of the FtsZ proteins from *Mycobacterium tuberculosis* (PDB: 1RLU) (Leung et al., 2004), *B. subtilis* (PDB: 2VAM), and *Pseudomonas aeruginosa* (PDB: 2VAW) (Oliva et al., 2007). The dimer model of EcFtsZ was obtained using the EcFtsZ monomer model and the crystal structure of *Methanocaldococcus jannaschii* FtsZ dimer structure (PDB: 1W59). These tasks were accomplished using the software Modeler version 9.6 (Sali and Blundell, 1993). The model with minimal energy structure was obtained performing molecular dynamics using the program NAMD 2.6 (Phillips et al., 2005). The dNAK 4 molecule (ligand) was constructed and geometrically optimized using the Gaussian package software (Gaussian 03, Revision C.01, Gaussian, Inc.: 2003). For control purposes, we performed a search of the binding sites via conformational sampling on the protein surface with the ICM program and the Pocket Finder module (An et al., 2005). The binding site was defined by a grid box of 15Å^3^ and both the lateral chains of the binding site and the ligands were allowed to be flexible. The docking procedure resulted in eight different conformations (poses) of the ligand in the binding site, scored with a calculated ΔG°. Each pose was further analyzed, discarding the poses in which the ligand appeared to be outside the site. Further optimization of the binding sites was performed, based on the molecular dynamics of the EcFtsZ dimer with GROMACS (Lindahl et al., 2001), to obtain different conformations, which were used for molecular docking. The docking free energy for the binding of the compound to each EcFtsZ conformation was used to select the best site. The hydrogen bonds between the compounds, and the groups of the amino acid residues in the binding site were determined and visualized using the computer program PyMol (Seeliger and de Groot, 2010).

## Results and Discussion

### Effect of dNAK 4 on the *in vitro* Polymerization of EcFtsZ and BsFtsZ

The structures of the derivatives of dihydroxynaphthyl ketones with different heterocycles (dNAKs) used in this work are shown in Table 1. The LogP and IFP values obtained for dNAK 4 indicate that this compound has the best solubility in water compared with the others, and presented the best inhibitory effect for EcFtsZ polymerization. For this reason, it was selected for further characterization as a potential antibiotic.

**Table 1:** Inhibition of EcFtsZ polymerization (IFP) and LogP for dNAKs 1–5.

**dNAK**	**Structure**	**IFP^**a**^ (μM)**	**Log P**
1	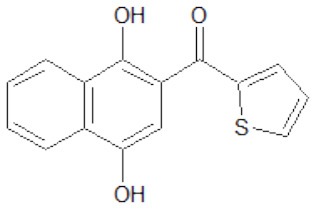	100^b^	3.9 ± 0.4
2	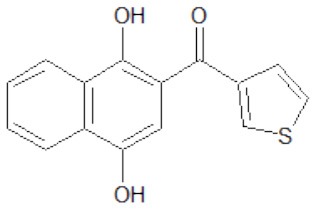	44^b^	3.9 ± 0.8
3	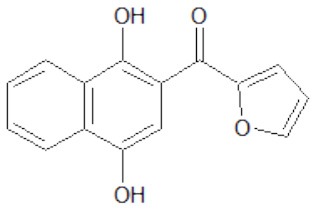	39^b^	3.0 ± 0.4
4	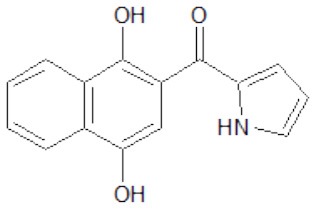	2.3 ± 0.1^c^ 4.4 ± 0.6^d^	2.4 ± 0.4
5	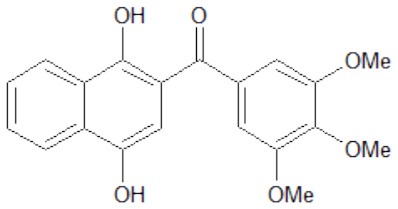	81.5^b^	4.3 ± 0.4

a *IFP is expressed as IC_50_ (μM)*.

b *IFP was calculated from the EcFtsZ polymerization inhibition by the compounds*.

c *IFP was calculated from EcFtsZ slope of light scattering increment produced by the compound after depolymerization. The PK/PEP GTP regeneration system was used (Margalit et al., 2004)*.

d *IFP was calculated from steady-state slope of light scattering increment produced by the compound. The PK/PEP GTP regeneration system was used*.

**Figure 1:**
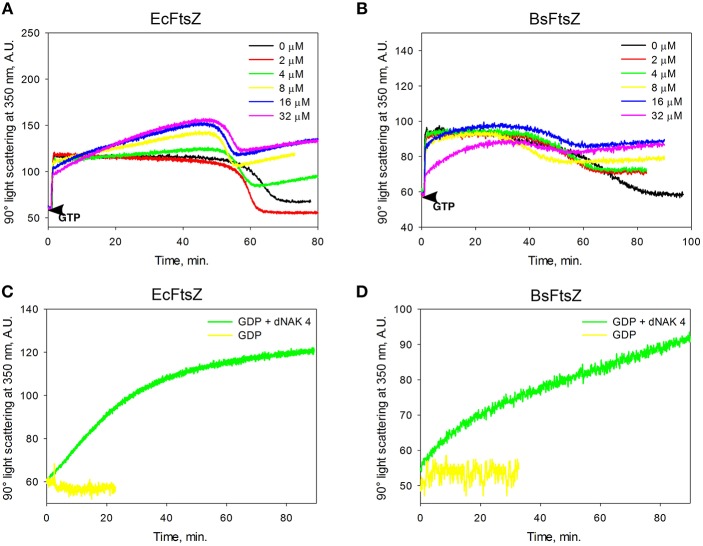
Effect of dNAK 4 on the *in vitro* polymerization of EcFtsZ and BsFtsZ. **(A,B)** show the GTP polymerization kinetics of EcFtsZ and BsFtsZ, respectively, at different concentrations of dNAK 4. **(C,D)** show the polymerization kinetics induced by GDP of EcFtsZ and BsFtsZ, respectively, in presence and absence of 32 μM dNAK 4. The protein concentration was 12.5 μM in a buffer containing 50 mM Hepes pH 7.3, 50 mM KCl, 10 mM MgCl_2_, and 1 mM GTP with PK/PEP regeneration system **(A,B)** or with 2 mM GDP **(C,D)**.

The EcFtsZ IFP value for dNAK 4 presented in Table 1 was determined from the results obtained in Figure 1A. The EcFtsZ polymerization curve in the absence of dNAK 4 presents three characteristic phases: a rapid initial polymerization, a steady state of the polymers, and depolymerization. In the presence of the compound, in the steady state part of the curve, there is a linear increase of the light scattering during the time, whose positive slope depends on the concentration of dNAK 4. A value of IC_50_ = 4.6 ± 0.6 μM was obtained from the steady-state slopes vs. dNAK 4 concentration (Table 1). On the other hand, the speed of depolymerization at the different dNAK 4 concentrations tested remained roughly unaffected. After depolymerization, the decrease of light scattering did not reach the same values compared to the control, with a linear increment in light scattering during the time that also depended on dNAK 4 concentration. A value of IC_50_ = 2.3 ± 0.1 μM was calculated from the slopes of the light scattering increment after depolymerization vs. dNAK 4 concentration.

**Figure 2:**
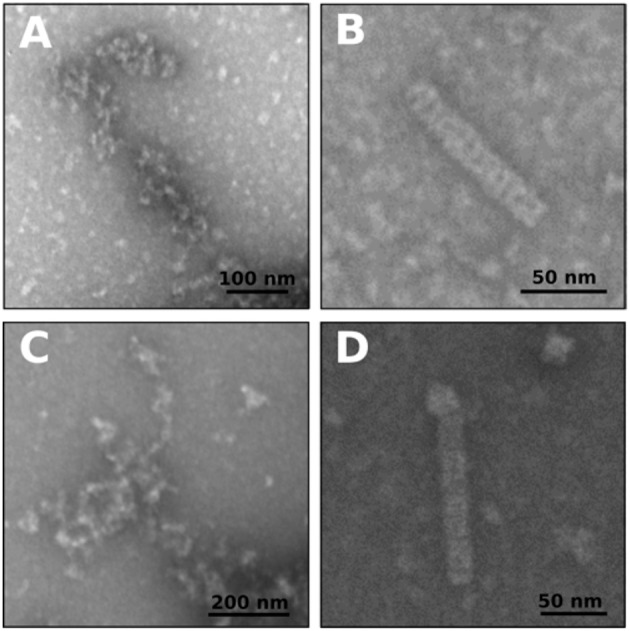
Negative staining electron microscopy of polymers of EcFtsZ and BsFtsZ with dNAK 4. **(A,B)** Electron microphotographs of filaments after depolymerization of GTP induced polymers of EcFtsZ and BsFtsZ, respectively, with 32 μM of dNAK 4. **(C,D)** Electron microphotographs of GDP induced polymers of EcFtsZ and BsFtsZ, respectively, with 32 μM of dNAK 4. In each case the protein concentration was 12.5 μM in a buffer containing 50 mM Hepes pH 7.3, 50 mM KCl, 10 mM MgCl_2_, and 1 mM GTP (and PK/PEP system) **(A,B)** or with 2 mM GDP **(C,D)**. A size bar is included in each figure.

The behavior of BsFtsZ after depolymerization was similar to that of EcFtsZ (Figure 1B). A value of IC_50_ = 9.13 ± 0.66 μM was obtained from the slopes of light scattering increment after depolymerization vs. dNAK 4 concentration. We interpret this behavior as the simultaneous polymerization of two classes of polymers, the formation of the usual GTP filaments that remain until depolymerization, and those obtained from a parallel polymerization induced by dNAK 4, which becomes evident after the GTP filaments depolymerization. Negative-staining electron microscopy analysis revealed that the formation of short curved filaments grouped in clusters and open rings that forms cylinders, exclusively with BsFtsZ-GDP (Figures 2B,D).

The increase of light scattering after the depolymerization of EcFtsZ and BsFtsZ observed with dNAK 4 is a phenomenon not described before, and occurs only with the GDP form of these proteins. In the absence of this compound, FtsZ normally remains as dimers and monomers.

To confirm the induction by dNAK 4 of FtsZ oligomers in the GDP conformation, we performed polymerization experiments with an excess of GDP. Figures 1C,D show the effect of GDP in the absence and in the presence of 32 μM dNAK 4, for EcFtsZ and BsFtsZ, respectively. The polymerization of both proteins was induced by the presence of dNAK 4. The kinetic behavior was similar to that shown in Figures 1A,B after the depolymerization of FtsZ induced by GTP. Polymerization of EcFtsZ-GDP and BsFtsZ-GDP depended on protein concentration, without a critical concentration value, and required magnesium.

### Morphology of EcFtsZ and BsFtsZ Filaments With dNAK 4

Figures 2A,B show electron micrographs of the polymers remaining after depolymerization of EcFtsZ and BsFtsZ GTP-induced filaments in the presence of 32 μM dNAK 4. Short and curved polymers of EcFtsZ were observed. In contrast, BsFtsZ formed small open-rings (diameter 18.9 ± 4.3 nm, *n* = 100), and cylinders (diameter 19.3 ± 2.4 nm, *n* = 37) probably made out from these rings. In the presence of GDP, both proteins formed polymers displaying a similar shape (Figures 2C,D). To our knowledge, cylinders of semi-rings induced by dNAK 4 are novel structures, and they, along with the semi-rings, are responsible for the polymerization inhibition of FtsZ.

The polymerization of EcFtsZ-GDP was previously described by Rivas et al. (2000). Our results indicate that dNAK 4 behaves as an agonist of EcFtsZ-GDP polymerization, while in the case of BsFtsZ, it stimulates lateral interactions of the open rings to form cylinders. To the best of our understanding, this behavior of BsFtsZ-GDP has not been previously described, and it is interesting because, on the one hand, it explains the inhibitory effect of dNAK 4 by the sequestration of active GDP-FtsZ and, on the other hand, supports the induction of lateral interactions between the open rings as the main force stabilizing the formation of the cylinders.

### Effect of dNAK 4 on the Growth of *E. coli* K12 and *B. subtilis*

**Figure 3:**
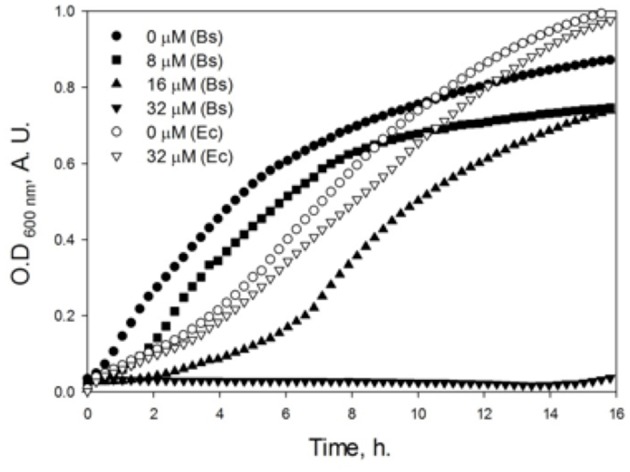
Effect of dNAK 4 on the growth of *E. coli* K12 and *B. subtilis*. Cultures of *E. coli* (empty symbols) and *B. subtilis* (filled symbols) were grown in Müller-Hinton media at 37°C, in the absence and with different concentrations of dNAK 4 indicated in the figure. The optical density at 600 nm was measured and recorded at different times.

In order to determine the inhibitory effect of dNAK 4 on the bacterial growth, the inhibition was measured in plates through the formation of growth inhibition halos on bacterial lawns (Table SM1), and indirectly through the characterization of the growth curves. Figure 3 shows that up to 32 μM dNAK 4 had no inhibitory effect on *E. coli* K12 growth in liquid culture. However, when this compound was tested in *B. subtilis*, there was an increase of the lag phase on the growth curves, with no growth at 32 μM during 16 h. The increase of the lag period depended on the concentration of dNAK 4, which is a typical bacteriostatic behavior (Walsh and Wencewicz, 2016), causing total growth arrest at the highest concentration used (32 μM). In plate assays, dNAK 4 showed better growth inhibition on *B. subtilis* with amounts as low as 0.1 μg (Table SM1). The inhibitory effect was also assessed on different Gram positive and negative strains. Thus, the same inhibitory effect in *B. subtilis* was observed in *S. aureus*, while the situation in Gram negative strains was different: no inhibitory effect was observed in *E. coli* and *E. aerogenes* at the concentration tested, whereas *P. aeruginosa* and *P. mirabilis* presented growth inhibition at 0.3–0.5 μg (Table SM1). The inhibitory effect was significant considering that, as proposed previously, a good antibiotic affects the bacterial growth with quantities of <4–8 μg when assayed in agar plates (García-Rodríguez et al., 2000). Moreover, dNAK 4 proved to be effective at a concentration of 32 μM (8 μg/mL), which is similar to the effective concentrations of commercial antibiotics, such as ampicillin or chloramphenicol (García-Rodríguez et al., 2000). All these properties make dNAK 4 a very good candidate as a starting molecule for the development of a new antibiotic, especially considering that this compound also inhibits the growth of bacterial strains such as *S. aureus* and *P. aeruginosa*, both species grouping pathogenic bacteria of clinical importance.

In order to evaluate the effect of dNAK 4 at a cellular level, we designed a live-cell imaging approach using the microfluidics platform CellASIC ONIX2 (Merck) coupled to a Lionheart FX automated microscope (BioTek). This setup allowed us to directly monitor the division of *E. coli* and *B. subtilis* cells inside microchambers, under a precisely controlled temperature (37°C) and the continuous perfusion of fresh culture medium. The microscope was set up to take one picture of each chamber every 10 min during the whole experiment (20 h), in order to get a continuous view of the division process occurring in a group of cells and its response to the perfusion of dNAK 4. First, cells of each strain were inoculated into separate microchambers, perfusing LB medium for 4 h while monitoring cell growth. Afterwards, LB supplemented with 32 μM dNAK 4 was perfused during the next 8 h. Finally, LB medium without dNAK 4 was perfused during the last 8 h in order to monitor the effect of washing the compound. Perfusion of dNAK 4 caused a complete growth arrest of *B. subtilis* cells (SM Movie 1), but had no effect on the growth of *E. coli* (SM Movie 2). After washing the compound, some *B. subtilis* cells seemed to partially recover the ability to divide. Unexpectedly, a very low level of filamentation was observed in *B. subtilis* cells, as consequence of the growth inhibition by dNAK 4. A plausible explanation for this behavior is that this compound has an additional inhibitory effect on cellular growth that does not rely on FtsZ function, possibly the production of reactive oxygen species (ROS). In this regard, it was demonstrated that naphtoquinones cause *M. tuberculosis* cell death through inducing NADH oxidation, ROS production, and possibly acting over other targets of the cellular redox metabolism (Heikal et al., 2016; Halicki et al., 2018).

Regarding the insensitivity of *E. coli* to dNAK 4, a probable explanation is that it cannot pass the outer membrane barrier. dNAK 4 has a LogP value of 2.41, which means that this compound has a poor solubility in hydrophobic media, likely impeding its diffusion through the outer membrane. If this is the case, the diffusion would depend on the particular composition of this membrane, because there are other Gram-negative bacteria such *P. aeruginosa* and *P. mirabilis* that are sensitive to the inhibitory effect of dNAK 4. Alternatively, the presence of particular porins in the outer membrane of the sensitive strains could be the pathway of entrance for this compound.

### dNAK 4 Binding Site in EcFtsZ

**Figure 4:**
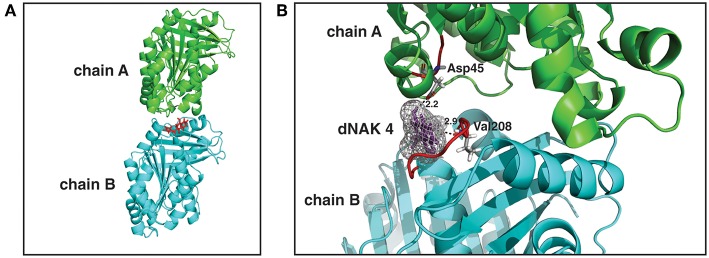
dNAK 4 binding site model in EcFtsZ dimer. **(A)** EcFtsZ model showing the structure of a dimer with the monomers is depicted in cyan and green, chain A and B, respectively, dNAK 4 is shown in red sticks. **(B)** Docking with EcFtsZ dimer of dNAK 4 (in violet sticks with its electronic density cloud in gray). Part of the secondary structure is indicated with the same colors used in **(A)** for each monomer. The sequence number of the amino acid residues involved in the interaction site is indicated and the loops colored in red. Dashed lines indicate hydrogen bonds and the numbers correspond to the distance in Angstroms.

We next sought to identify the binding site of dNAK in the FtsZ dimer. To this end, a model of the EcFtsZ monomer was first generated using the amino acid sequence of EcFtsZ and the crystal structures of the FtsZ proteins from *M. tuberculosis, B. subtilis*, and *P. aeruginosa*. Then, a model of the EcFtsZ dimer was obtained using the EcFtsZ monomer model and the crystal structure of *M. jannaschii* FtsZ dimer structure. Molecular docking calculations between dNAK 4 and the FtsZ dimer model indicated that the binding site is located at the surface of the interface between the two monomers. Specifically, it resides in a hydrophobic pocket located between the amino domain of one monomer and the carboxyl domain of its neighbors in the polymer (Figures 4A,B). In this site, the carbonyl group of Asp45, part of a peptide bond in loop T2 of one monomer of the dimer, interacts with the compound through a hydrogen bond with the hydroxyl group at the carbon 1 (C-1) of the naphtyl group. Also, the carbonyl group of Val208 of the peptide bond, located in loop T7 of the other monomer, interacts through a hydrogen bond with the nitrogen of the pyrrol group of dNAK4. The calculated distance between the donor and acceptor of the hydrogen bond is 2.2 and 2.6Å, respectively. The conformation of the compound in this site was found to be the most thermodynamically favorable. To assess the importance of the dNAK 4 naphtyl group for the binding to FtsZ, a comparison of the free energy of binding of dPAK 4, a molecule that has a phenyl instead of a naphtyl group, was performed. The free energy was determined using the computer program Autodock Vina (Trott and Olson, 2010), and the values for the binding of dPAK 4 and dNAK 4 to EcFtsZ were 6.5 and 7.5 kcal/mol, respectively, indicating that the presence of two rings in the naphtyl group favored the binding of dNAK 4. This difference of 1 kcal/mol can be explained by the higher hydrophobic effect of the naphtyl group compared to that of the phenyl group, as we confirmed using the computer program LigandScout (Wolber and Langer, 2005). Additionally, we corroborated that the naphtyl group is essential for dNAK 4 effect on FtsZ polymerization, since dPAK 4 showed no inhibitory effect over Ec and BsFtsZ polymerization (Figures SM 1A,B).

### Mechanism of Semi-rings and Cylinders Formation Induced by dNAK 4

**Figure 5:**
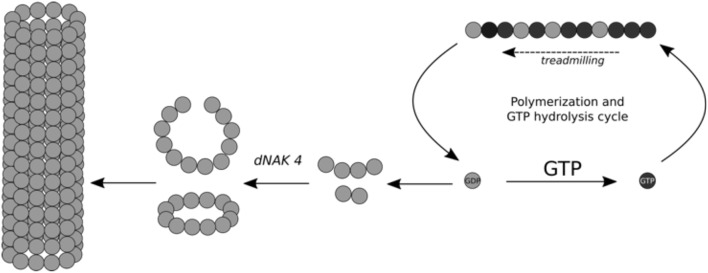
Scheme for the mechanism of open rings and cylinders formation induced by dNAK 4. Schematic representation of dNAK 4 effect on BsFtsZ polymerization. The filaments induced by GTP to explain the mechanism of inhibition of the polymerization of BsFtsZ produced by dNAK 4 is included. BsFtsZ-GDP form is indicated with gray spheres and the BsFtsZ-GTP form with black spheres. The scheme shows cylinder formation induced by GDP and the loss of protein suitable for filament formation, due to “sequestration” into the cylinders and their precursors.

Based on the results shown in this study, we generated a model explaining the effect of dNAK4 on the formation of FtsZ polymers, which is summarized in Figure 5. The mechanism of BsFtsZ polymerization induced by GTP is shown at the right of the scheme. Hydrolysis of GTP and addition of new GTP-FtsZ monomers produce treadmilling of the filament (Bisson et al., 2017; Yang et al., 2017). The GDP-FtsZ monomers exchange GDP by GTP and the cycle of polymerization continues. Part of GDP-FtsZ forms small curved filaments that, in the presence of dNAK 4, are stabilized and new GDP-FtsZ monomers are added to form small semi-rings of 12 subunits. These semi-rings interact laterally to form cylinders of different length. Notwithstanding, many semi-rings remain in solution, probably due to a high critical concentration for the formation of the cylinders.

Finally, it is interesting to remark that dNAK 4 is a very good inhibitor of FtsZ polymerization because it binds to the GDP conformation of FtsZ. The affinity of this binding is better than some antibiotics that are currently in use, and the fact that it does not affect eukaryotic cells in the range of its inhibitory concentrations (Benites et al., 2016) makes this compound a very good antibiotic candidate.

## Conclusions

dNAK 4 showed a strong inhibitory effect on *in vitro* Ec and BsFtsZ polymerization, which occurs in the same micromolar range to inhibition of tubulin polymerization (Gutiérrez et al., 2015). *In vivo*, it was a very effective inhibitor of *B. subtilis* growth, but had very little effect over *E. coli*. The FtsZ inhibition mechanism of dNAK 4 is different to that described for other FtsZ-targeting compounds and is dependent on the presence of its naphtyl group. It does not interact with GTP-FtsZ in the monomeric and polymeric state, but rather, it has increased affinity for GDP-FtsZ in polymeric state, favoring the formation of curved small filaments. This process depends on magnesium and protein concentration, without a critical concentration (isodesmic polymerization). Therefore, the inhibition of FtsZ polymerization should be produced by an indirect sequestering effect of the GDP-FtsZ forms in oligomeric state, as non-functional polymers. In agreement with this, polymerization induced by GTP, in the presence of dNAK 4, maintained a similar critical concentration.

## Data Availability

The raw data supporting the conclusions of this manuscript will be made available by the authors, without undue reservation, to any qualified researcher.

## Author Contributions

OM and RL conceived and designed the experiments. JB and JV performed the synthesis and characterization of the compounds. GA, JR, and AM performed the experiments. OM, RL, and GA wrote the paper. All authors reviewed the manuscript.

### Conflict of Interest Statement

The authors declare that the research was conducted in the absence of any commercial or financial relationships that could be construed as a potential conflict of interest.
