# Supplementary material for: Inhibition of Escherichia coli and Bacillus subtilis FtsZ Polymerization and Bacillus subtilis Growth by Dihydroxynaphtyl Aryl Ketones
Source: Front Microbiol. 2019 Jun 12;10:1225. doi: 10.3389/fmicb.2019.01225 (PMC6582257; doi:10.3389/fmicb.2019.01225)
Supplement: Supplementary file 3 [file Data_Sheet_1.pdf]

Inhibition of *Escherichia coli* and *Bacillus subtilis* FtsZ polymerization and *Bacillus subtilis* growth by dihydroxynaphtyl aryl ketones.

Gissela Araya, Julio Benites, Juan S. Reyes, Andrés Marcoleta, Jaime A. Valderrama, Rosalba Lagos and Octavio Monasterio.

Supplementary Material

Effect of dPAK 4 [(2,5-dihydroxy-phenyl)-(1*H*-pyrrol-2-yl)-methanone] on the *in vitro* polymerization of EcFtsZ and BsFtsZ. Figure SM1A shows that in the presence of dPAK 4, the steady state part of the curve of EcFtsZ polymerization has the same shape than the control, lacking the linear increase over time of the light scattering observed with dNAK 4 (Fig. 1 A and B). However, there was an increment of the overall light scattering level at the steady state (without altering the shape of the curve) at the two highest concentrations of dPAK 4 tested. This effect was also observed in BsFtsZ polymerization (figure SM1B), but at lower dPAK 4 concentrations. After depolymerization, the light scattering level remained constant over time, with a shape that resembles a normal depolymerization such as that observed for the control. These results indicate that the GDP-FtsZ form is not able to polymerize in the presence of dPAK 4. Based on this evidence, we propose that one of the rings of the naphtyl group, specifically that without the hydroxyl groups, is crucial for the increment of the slope at the steady state produced by polymerization of the FtsZ-GDP in the presence of dNAK 4. This conclusion is in agreement with the free energy of binding calculated by bioinformatics, which favor the binding of dNAK 4 to FtsZ by 1 kcal/mol.The structure of dPAK 4 is shown in the figure SM1 A.

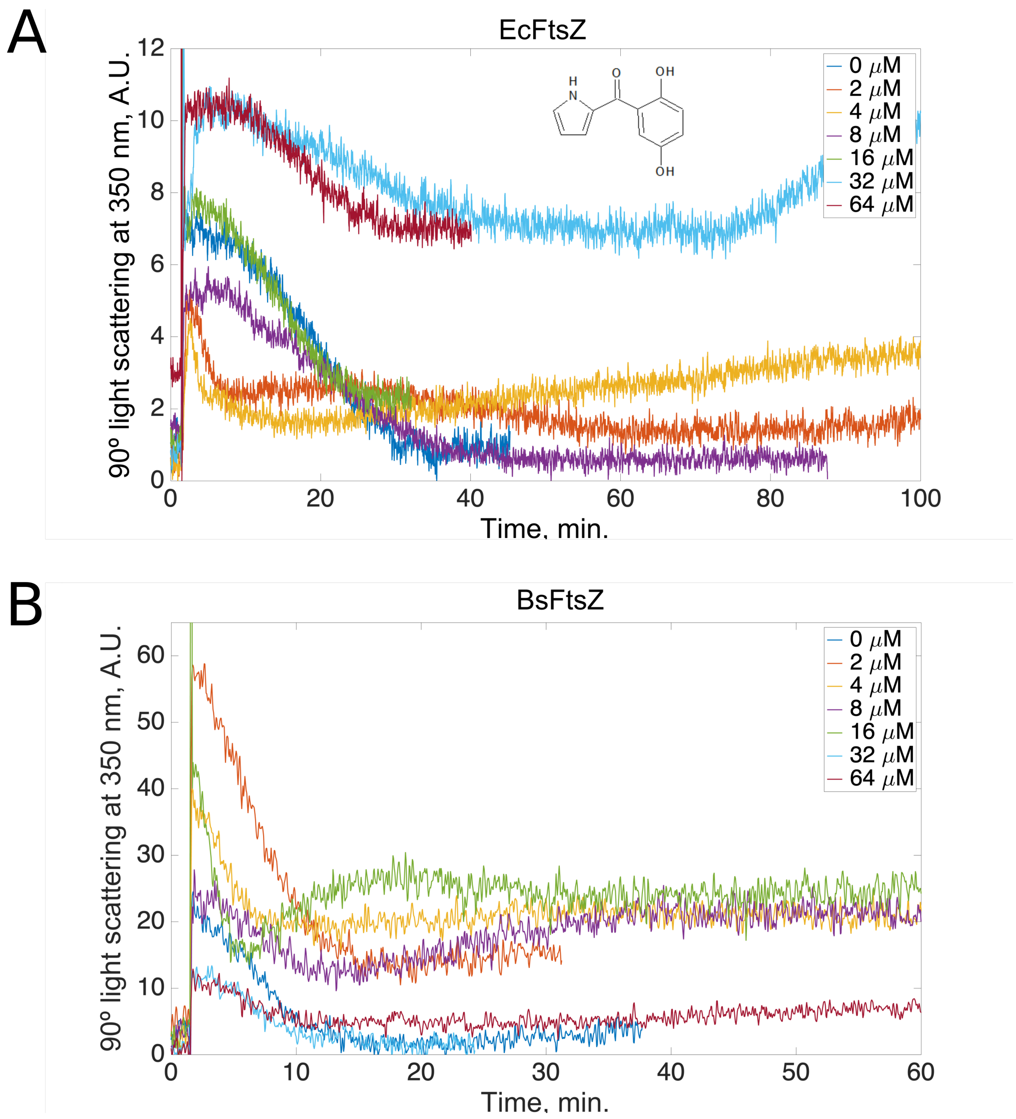

Figure SM 1. FtsZ polymerization curves in presence of increasing concentrations of dPAK 4. A) EcFtsZ with 2, 4, 6, 8, 16, 32 and 64 μM dPAK 4 with 1% DMSO in the polymerization buffer. B) BsFtsZ with 2, 4, 6, 8, 16, 32 and 64 μM of dPAK 4 with 1% DMSO in the polymerization buffer (see Materials and Methods). The structure of dPAK 4 is included in A.

TABLE SM1. Effect of dNAK 4 tested directly on the growth of different bacterial lawns

Strains	Amount of dNAK 4 in DMSO 30%					
Gram (–)	**0.05 μg**	**0.1 μg**	**0.3 μg**	**0.5 μg**	**1 μg**	**2 μg**
Enterobacter aerogenes BEM27	–	–	–	–	–	–
Escherichia coli K12	–	–	–	–	–	–
Pseudomonas aeruginosa BEM12	–	–	+	+	+	+
Proteus mirabilis BEM87	–	–	–	+	+	+
Gram (+)						
Bacillus subtilis BEM54	–	+	+	+	+	+
Staphylococcus aureus BEM42	–	+	+	+	+	+

Absence (-) or presence (+) of growth inhibition halos on different bacterial lawns. The bacterial lawns for each strain listed in this Table were prepared as follow: Bacterial cells were grown in Müller-Hinton (MH) media until an OD_{600} = 0.3. 300 μl of each culture was mixed with 5 ml of MH soft agar and poured into a plate containing 20 ml of MH hard agar. Once cooled, 5 μl of dNAK 4 dissolved in DMSO 30% (freshly prepared) containing the amount indicated in the table were placed on the top agar and incubated at 37°C during 5-6 h until growth inhibition halos were observed. As negative control, 5 μl of 30% DMSO was used, which did not produce growth inhibition halos in any of the strains tested. As positive control the antibiotic minocycline was used. The experiments were performed by triplicate. The bacterial strains used in these experiments belong to our laboratory collection.

2. Live-cell imaging of *E. coli* and *B. subtilis* cell division while grown in microfluidic chambers, in the presence or absence of dNAK 4.

The effect of dNAK 4 over the bacterial growth was investigated at a cellular level and in a dynamic fashion, monitoring by live-cell imaging the cell division of *E. coli* and *B. subtilis* in a microchamber, using the microfluidics platform CellASIC ONIX2 (Merck), allowing the programmable perfusion of fresh medium with or without dNAK 4. The experiment started inoculating each strain in a microchamber and then start perfusing LB medium and during 4 h. Afterwards, LB supplemented with 32 μM dNAK 4 was perfused during 8 hours, and finally the compound was washed perfusing LB medium alone during 8 hours. The monitoring of the cells during the whole perfusion scheme and the technical details of the CellASIC ONIX2 setup are described as supplementary methods. As shown in Supplementary Movies 1 and 2, perfusion of dNAK4 caused a complete growth arrest of *B. subtilis* cells, but had no effect on the growth of *E. coli*.

Supplementary methods

Live-cell imaging of bacterial cell division in a CellASIC ONIX2 microfluidics chamber.

Bacterial strains were grown overnight in LB medium at 37°C. The next day in the morning, the cultures were used to inoculate fresh medium (1/20) and grown to exponential phase (D.O. 600 nm: 0.4-0.8). Subsequently, bacterial cells were harvested by centrifuging at 4.000 rpm during 5 min, and then resuspended in LB, concentrating them 10X. 50 μL of each concentrated cell suspension were loaded into a B04 plate of the CellASIC ONIX2 microfluidics platform (Merck), following the manufacturer's

guidelines. After cell loading, the following perfusion scheme was programmed (keeping the temperature at 37 ± 0.2 °C during the whole experiment): 4 h of LB medium, then 8 h of LB supplemented with 32 μM dNAK 4, then 8 h with LB alone. The bright-field imaging of the microfluidics plate was performed in a Lionheart FX automated microscope (BioTek), set up to take pictures every 5 minutes, during a total period of 20 h, using a 60X air objective. Imaging setup, image capture and processing, as well as movie rendering was performed using the Gen5 software (BioTek).
